# Digital Analysis of Dentogingival Factors Influencing the Gingival Margin Position in the Esthetic Zone

**DOI:** 10.1111/jerd.13407

**Published:** 2025-01-08

**Authors:** Hesham H. Abdulkarim, Bryan Habet, Alexander G. Ahmadi, Mary Ying‐Fang Wang, Elio Reyes Rosales, D. Douglas Miley

**Affiliations:** ^1^ Missouri School of Dentistry & Oral Health, Department of Advanced Periodontics & Dental Implant Care A.T. Still University Saint Louis Missouri USA; ^2^ Department of Periodontics, Center of Advanced Dental Education Saint Louis University, Private practice Chicago Illinois USA; ^3^ Department of Research Support A.T. Still University Kirksville Missouri USA; ^4^ Center of Advanced Dental Education, Department of Periodontics Saint Louis University Saint Louis Missouri USA

**Keywords:** alveolar bone, cone beam computed tomography, esthetic crown lengthening, gingival phenotype

## Abstract

**Objectives:**

To investigate the correlation between gingival thickness (GT) and buccal bone thickness (BBT), as well as the effects of GT, BBT, bone crest level (BC), and tooth position on the buccal gingival margin location of maxillary teeth in the esthetic zone.

**Materials and Methods:**

Periodontally healthy subjects with prior cone beam computed tomography and intraoral scans for dental implant planning were included. The hard and soft tissue measurements were retrospectively analyzed digitally. Pearson's correlation and ANOVA were used to evaluate the correlations between clinical/anatomical crown height ratios (CCH/ACH), gingival and buccal bone thicknesses, and the distance between the CEJ and BC (CEJ‐BC).

**Results:**

Forty‐two subjects with 252 teeth were analyzed. A positive correlation was found between GT and BBT (*p* < 0.001). GT and BBT were negatively correlated with CCH/ACH (*p* = 0.006 and *p* = 0.004, respectively), while CCH/ACH showed a positive correlation with CEJ‐BC (*p* < 0.001).

**Conclusions:**

Within the limitations of our study, we concluded that gingival and buccal bone thicknesses were positively correlated. A thicker gingival phenotype and higher coronal bone crest were linked to a more coronal gingival margin in the esthetic zone.

## 1 Introduction

The gingival tissue contour plays a vital role in smile esthetics, especially in the maxillary anterior region [1]. The position of the gingival margin on the crown is crucial as it determines clinical crown height, defined as the distance from the deepest concavity of the gingival margin to the incisal edge or occlusal surface [2].

Individual variations in clinical crown height were documented, with potential influences from gender and race [3, 4]. G. V. Black also highlighted age‐related changes, noting that the gingival length from the line of attachment to the tooth neck varies across individuals. He observed that this measurement is often greater in younger individuals and diminishes with age [5].

Gottlieb and Orban later described the age‐related change in gingival position as passive eruption. They defined active eruption as the movement of a tooth toward the occlusal plane, whereas passive eruption refers to the movement of the gingival margin toward the tooth apex [6]. Goldman and Cohen described Altered Passive Eruption (APE) as a failure of the gingival margin to recede to a level apical to the cervical convexity of the crown during tooth eruption [7]. Coslet et al. proposed a classification of APE based on keratinized gingiva dimensions and the relationship of the alveolar bone crest (BC) to the cementoenamel junction (CEJ) [8]. In 2017, Ragghianti Zangrando et al. proposed an updated classification where they denoted a difference in APE and Altered Active Eruption (AAE) [9].

APE is a finding usually given when the free gingival margin is more than the normal 0.5‐2 mm coronal to CEJ position as described by Ainamo and Loe [10]. Coslet et al. and Ragghianti Zangrando et al. describe APE Type 1 as having a keratinized tissue width of > 2 mm and Type 2 as having a keratinized tissue width < 2 mm. A CEJ‐BC distance of > 1.5 mm is termed subgroup A by Coslet et al., while no additional terminology is added in this case by Ragghianti Zangrando et al. However, a CEJ‐BC distance of < 1.5 mm is termed subgroup B by Coslet et al. and described as AAE by Ragghianti Zangrando et al. [8, 9].

The periodontal soft‐tissue phenotype significantly influences the long‐term stability of the gingival margin [11]. Individuals with a thinner gingival phenotype and thinner buccal bone below the CEJ are more susceptible to gingival recession than those with a thicker gingival phenotype and thicker bone [12, 13]. This highlights the role of both gingival thickness and buccal bone in determining the stability and position of the gingival margin, as well as the clinical crown height.

Several studies have evaluated the value of CBCT in assessing soft and hard tissue thickness. CBCT has confirmed its accuracy in evaluating the gingival phenotype [14, 15], the relationship between the BC, CEJ, and gingival margin [16, 17], and confirmed the positive correlation between gingival phenotype and buccal bone thickness (BBT) [17, 18, 19, 20, 21]. This makes it a valuable tool for diagnosis and treatment planning. In addition to the standalone use of CBCT, combining intraoral scans with CBCT by superimposing the resultant files effectively highlights the location of soft tissue relative to the underlying bone. This combined technique offers accurate soft tissue visualization [17, 22, 23], and enables the evaluation of post‐operative soft tissue stability [22].

Accepted definitions and expectations for active and passive eruption during growth have been previously described [6, 7, 10]. Despite the availability of existing APE classification systems and proposed treatment approaches [8, 9], the relationships between teeth, soft tissue, and bone that contribute to the development of APE cases observed in middle to late adulthood remain unclear. A deeper understanding of the biological and anatomical factors influencing gingival margin position is essential for achieving optimal esthetic treatment outcomes.

The objectives of this study were to investigate the correlation between gingival thickness (GT) and BBT. It also aimed to assess the impact of GT, BBT, bone crest level (BC) relative to the cementoenamel junction (CEJ), and tooth position on the location of the labial/buccal gingival margins of maxillary teeth in the esthetic zone, using a non‐invasive digital method.

## 2 Materials and Methods

### 2.1 Ethical Approval

This retrospective investigation was approved by A. T. Still University Institutional Review (IRB) in December 2021 (IRB NA20221103‐001).

### 2.2 Eligibility Criteria and Recruitment

Patients who sought dental implant treatment planning at the Department of Advanced Periodontal and Dental Implant Care at the Department of Advanced Periodontal and Dental Implant Care at Missouri School of Dentistry & Oral Health between October 2018 and March 2024 were eligible for analysis if they had undergone CBCT scan and Intraoral Scan (IOS) for treatment‐related purposes. All patients included in the study signed written informed consent for their treatment.

Exclusion criteria included the following: (1) pregnant patients, patients with uncontrolled systemic disease, or patients taking medications which are known to cause gingival enlargement; (2) patients with clinical or radiographic signs of periodontal disease; (3) patients with a history of scaling and root planning or periodontal treatment; (4) patients with clinical or radiographic tooth abnormalities; (5) malpositioned, rotated, intruded or extruded teeth; (6) patients whose CBCT scan showed excessive scatter or inability to accurately identify the position of the incisal edge/cusp tip, free gingival margin (FGM) and/or CEJ (e.g., teeth with crown restorations, cervical restorations or incisal edge/cusp tip restorations); (7) patients with incomplete IOS that did not capture the area of interest; (8) teeth with signs of recession (FGM) apical to CEJ either clinically or after scan registration in a digital imaging viewing software (Blue Sky Plan, BlueSkyBio LLC); (9) patients with excessive tooth wear (clinically visible dentin or obliterated incisal or occlusal enamel seen after scan registration); and (10) patients without the minimum requirement of two measurable teeth on combination scan.

### 2.3 STL and CBCT Image Acquisition and Superimposition

**FIGURE 1:**
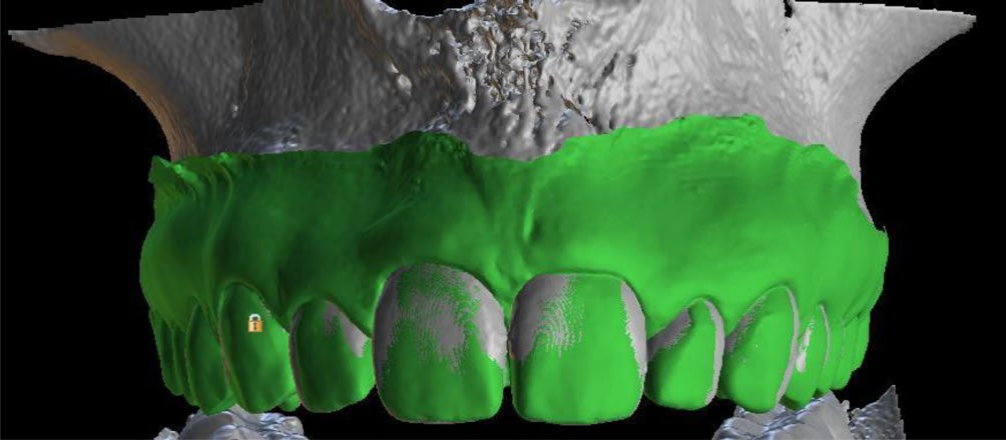
Combination scans with STL overlaid onto 3D reconstruction of DICOM data.

All subjects planned for an endosseous dental implant in the esthetic zone, who received a pre‐operative CBCT (CS 8100, Carestream Dental) and intraoral scan (IOS) (Cerec Omnicam, Dentsply Sirona) for treatment planning, were considered. The CBCT image was exported as in DICOM format, while the IOS was exported in STL format. The STL model was then superimposed onto the DICOM files using a best‐fit algorithm with digital imaging software (Blue Sky Plan, BluSkyBio LLC), as described by Abdulkarim et al. [22] The superimposition was first automatically done via the software, then manually adjusted for optimal fit to ensure accurate measurements before data collection (Figure 1).

### 2.4 Clinical and Image Analysis

Every maxillary tooth from right second premolar to left second premolar with an accurately aligned combination scan was evaluated. Teeth were sub‐grouped based on tooth type as central incisor (CI), lateral incisor (LI), canine (CA), first premolar (P1), and second premolar (P2).

**FIGURE 2:**
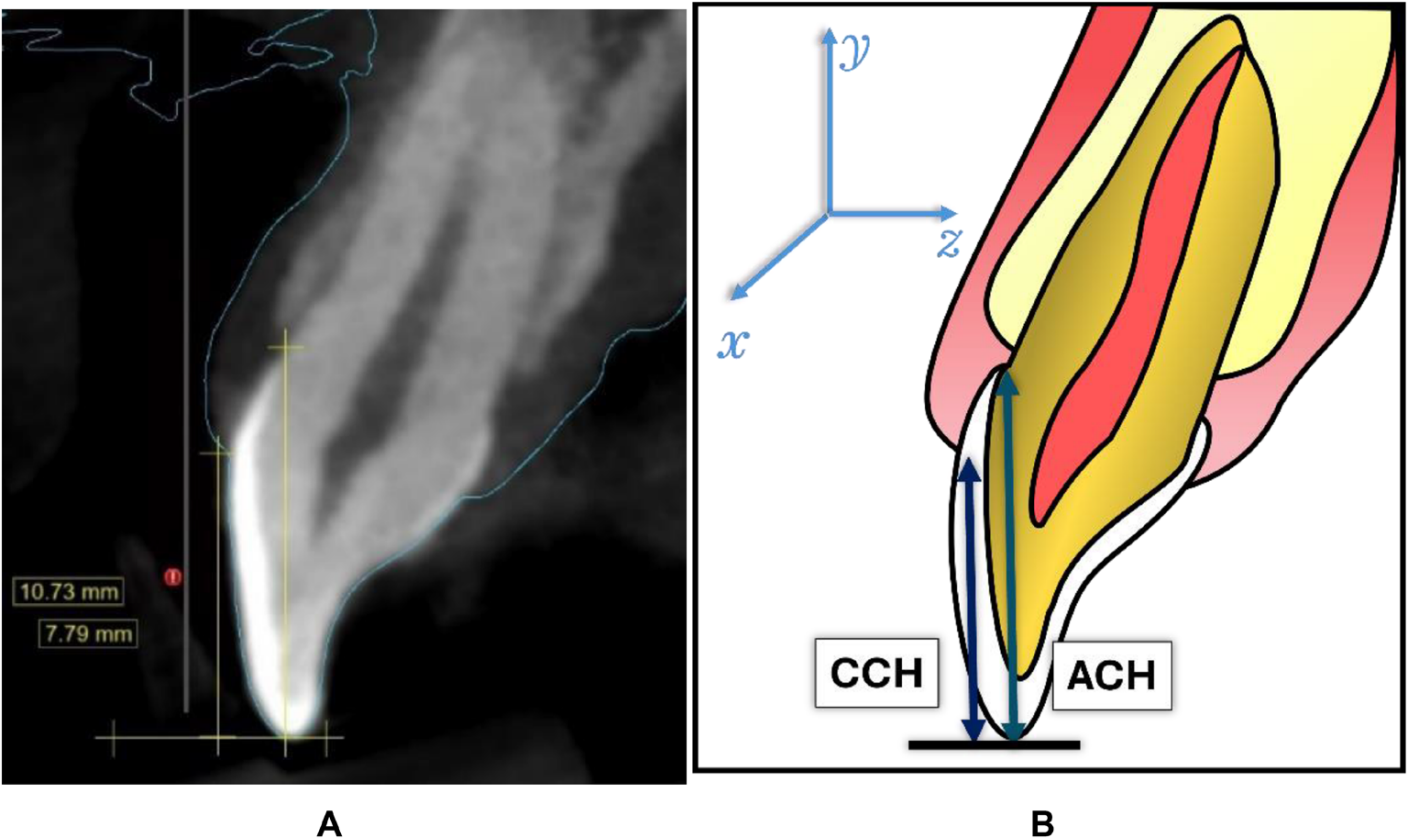
(A) Sagittal section of a central incisor displaying the measurements of CCH and ACH on a CBCT overlaid with an STL file using digital imaging software (Blue Sky Plan, BlueSkyBio LLC). (B) Diagram depicting CCH and ACH with the alignment of axes for measurements. ACH: anatomical crown height. CCH: clinical crown height.

Two sets of measurements were obtained. First, using the digital image viewing software (Blue Sky Plan, BluSkyBio LLC), the sagittal section of each tooth was evaluated by centering the *y*‐axis on the longest apico‐coronal distance of each tooth's anatomic crown and the *x*‐axis on the longest corresponding mesio‐distal distance of each tooth's anatomic crown. The 0 point of the *y*‐ and *z*‐axes was set to the incisal edge/cusp tip. Measurements recorded along the Y‐axis included the following: (1) from CEJ to incisal edge/cusp tip for the anatomic crown height (ACH) and (2) from FGM to incisal edge/cusp tip for the clinical crown height (CCH) (Figure 2). The clinical crown height/anatomic crown height ratio (CCH/ACH) was calculated. The CCH to ACH ratio (CCH/ACH) was used to adjust for anatomical crown size. A higher CCH/ACH ratio signified a more apical gingival margin, indicating a larger clinical crown relative to the anatomical crown, and vice versa. Clinical and anatomical crown measurements were captured and recorded by a single calibrated examiner, BH, with (0.99) intra‐class correlation for CCH and ACH on repeated measurements of 26 sites each.

**FIGURE 3:**
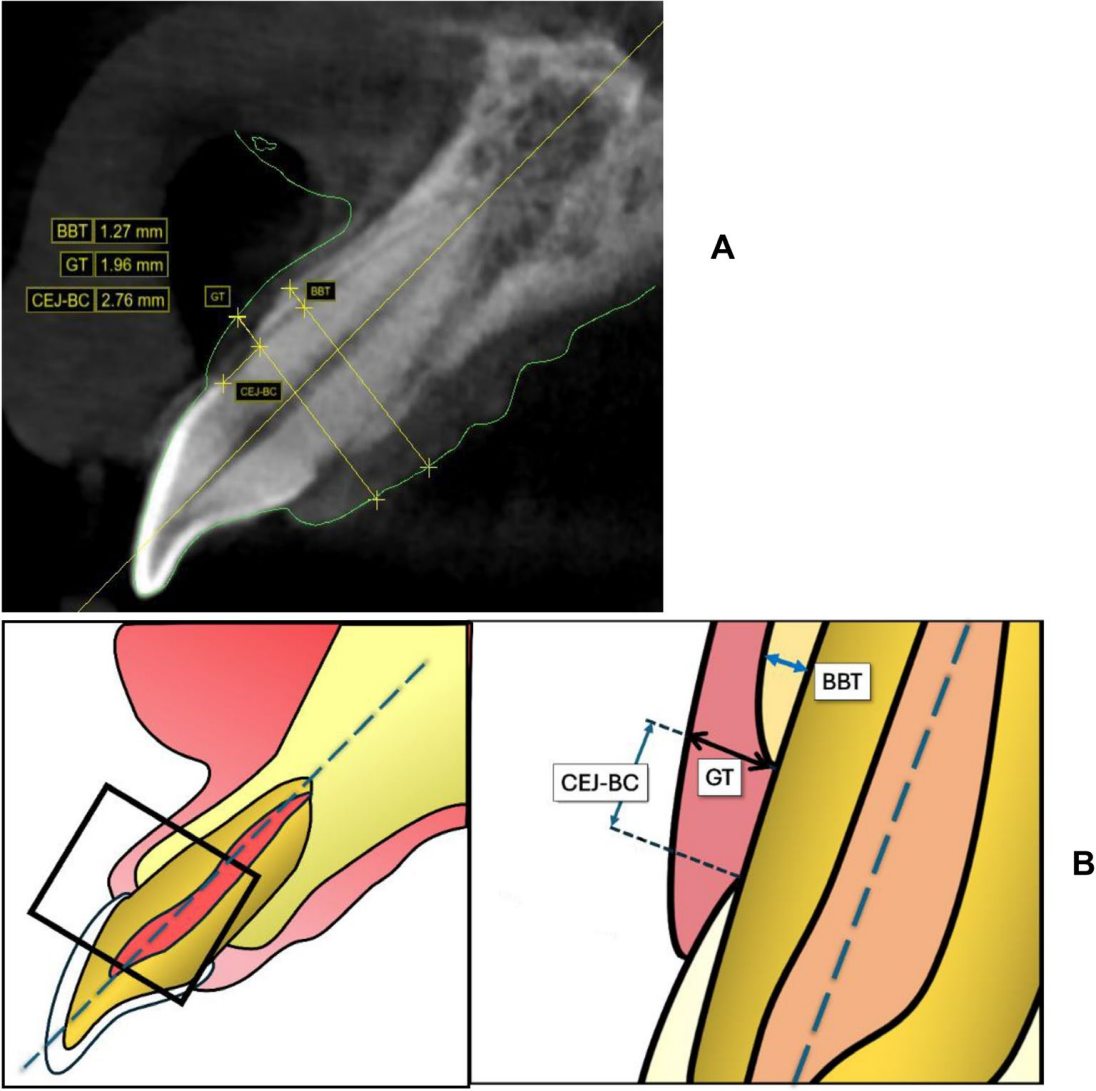
(A) Sagittal section of a central incisor illustrating the captured measurements on a CBCT, overlaid with an STL file using digital imaging software (Blue Sky Plan, BlueSkyBio LLC). (B) Diagram of a magnified sagittal section of the maxillary central incisor, showing landmarks and measurements. BBT: buccal bone thickness. BC: bone crest. CEJ: cementoenamel junction. GT: gingival thickness.

Second, the sagittal section of each tooth was evaluated by centering the *y*‐axis on the longest apico‐coronal distance of each tooth and the *x*‐axis on the longest mesio‐distal aspect of each tooth perpendicular to the buccal bone. Measurements recorded along the Z‐axis included the following: (1) distance from the CEJ to the BC (CEJ‐BC); (2) distance from gingival margin to bone crest (FGM‐BC); (3) gingival thickness (GT) measured at the level of the alveolar crest; (4) thickest buccal bone (BBT) measured at the thickest point of labial/buccal bone in the coronal half of the root (Figure 3). These measurements were captured and recorded by a single calibrated examiner, HA, with (0.99) intra‐class correlation for FGM‐BC on repeated measurements of 44 sites.

The crown was the reference for the first measurements while the tooth in whole was used as the reference for the second measurements for the purpose of calibration.

### 2.5 Statistical Analyses

For all measurements, sample mean, standard deviation (SD), and range were calculated for each tooth position, as well as for all teeth combined without adjusting for the different sample size of each tooth position One‐way ANOVA was used to detect a global difference due to tooth positions for the following measurements: (1) GT, (2) BBT, and (3) CCH/ACH. When a global difference is present, post hoc t‐tests with Bonferroni correction were conducted to compare tooth positions in a pairwise manner. Two independent sample t‐tests were used to compare GT and BBT among genders. Pearson's correlation coefficient was used to explore the relationship between (1) GT versus all other measurements and age, (2) BBT versus all other measurements and age, and (3) CCH/ACH versus CEJ‐BC. All statistics were obtained using SPSS, Version 29.0, and a *p*‐value (< 0.05) was considered statistically significant.

### 2.6 Sample Size Calculation

At a significance level of 0.05 and 80% power, approximately 193 teeth are needed to detect a true Pearson correlation coefficient that is greater than 0.2 or less than −0.2. To detect a difference among CI, LI, CA, P1, and P2, ~29 of each tooth type are needed if the true difference is about the same as SD. PASS, Version 13, was used for the sample size calculation.

## 3 Results

A total of 201 implant patients were screened for inclusion, with the majority excluded due to incomplete IOS that did not capture sufficient soft tissue. Additional exclusions were made based on other criteria. Ultimately, 42 cases were included. Of the teeth from the included subjects, 26 were excluded: 13 due to restorations affecting the incisal edge and/or CEJ, 6 due to recession, 6 due to difficulty aligning the CBCT and IOS, and 1 due to being a peg lateral. The study included 252 teeth from 42 subjects, consisting of 31 females and 11 males, with an average age of 44.5 years (SD = 15.2). Of the 252 teeth, 142 were from subjects aged 19–39, 77 from subjects aged 40–60, and 33 from subjects aged over 61.

The average number of teeth with measurements for each patient at CI, LI, CA, P1, and P2 were 1.5, 1.2, 1.3, 0.8, and 1.1, respectively. Altogether, 252 teeth (62 CI, 52 LI, 56 CA, 34 P1, and 48 P2) were measured. The mean and range of each measured dimension is shown in (Table 1), for all teeth combined and for each tooth position.

**TABLE 1:** Means and ranges of each dentogingival measurement, overall and by tooth position, recorded in millimeters.

Measurement	Overall^a^			CI			LI		
	*N*	Mean (SD)	Range	*N*	Mean (SD)	Range	*N*	Mean (SD)	Range
CCH	252	8.38 (1.52)	4.84–11.95	62	9.67 (1.09)	7.38–11.95	52	8.12 (1.22)	5.15–10.34
ACH	252	9.94 (1.52)	6.75–13.05	62	11.43 (0.90)	9.56–13.05	52	10.03 (0.85)	8.26–11.77
CCH/ACH	252	0.84 (0.08)	0.57–0.99	62	0.85 (0.07)	0.69–0.99	52	0.81 (0.09)	0.57–0.96
CEJ‐BC	252	2.17 (0.83)	0.26–5.35	62	2.34 (0.97)	0.50–5.35	52	2.33 (0.68)	1.21–4.17
BBT	252	1.30 (0.65)	0.28–3.50	62	1.00 (0.41)	0.29–2.23	52	1.02 (0.39)	0.28–2.33
GT	252	1.67 (0.52)	0.68–3.64	62	1.52 (0.41)	0.68–2.72	52	1.48 (0.35)	0.76–2.62

Measurement	CA			P1			P2		
	N	Mean (SD)	Range	*N*	Mean (SD)	Range	*N*	Mean (SD)	Range
CCH	56	9.13 (1.19)	7.01–11.76	34	7.37 (0.74)	5.98–9.46	48	6.81 (0.98)	4.84–8.37
ACH	56	10.69 (0.97)	8.55–12.23	34	8.45 (0.63)	7.48–10.15	48	8.11 (0.76)	6.75–9.77
CCH/ACH	56	0.85 (0.07)	0.66–0.99	34	0.87 (0.06)	0.73–0.99	48	0.84 (0.08)	0.69–0.97
CEJ‐BC	56	2.22 (0.84)	0.66–4.62	34	2.13 (0.73)	0.64–3.40	48	1.77 (0.72)	0.26–3.07
BBT	56	1.17 (0.51)	0.37–2.61	34	1.40 (0.62)	0.48–2.84	48	2.05 (0.71)	0.91–3.50
GT	56	1.57 (0.52)	0.70–3.34	34	1.84 (0.46)	1.11–2.80	48	2.08 (0.58)	1.01–3.64

^a^ The overall mean without adjusting for the different sample size of each position.

The mean CCH for CI, LI, CA, P1, and P2 was found to be 9.67 ± 1.09 mm, 8.12 ± 1.22 mm, 9.13 ± 1.19 mm, 7.37 ± 0.74 mm and 6.81 ± 0.98 mm. The mean ACH for CI, LI, CA, P1, and P2 was found to be 11.43 ± 0.90 mm, 10.03 ± 0.84 mm, 10.69 ± 0.97 mm, 8.45 ± 0.63 mm, and 8.11 ± 0.76 mm.

**FIGURE 4:**
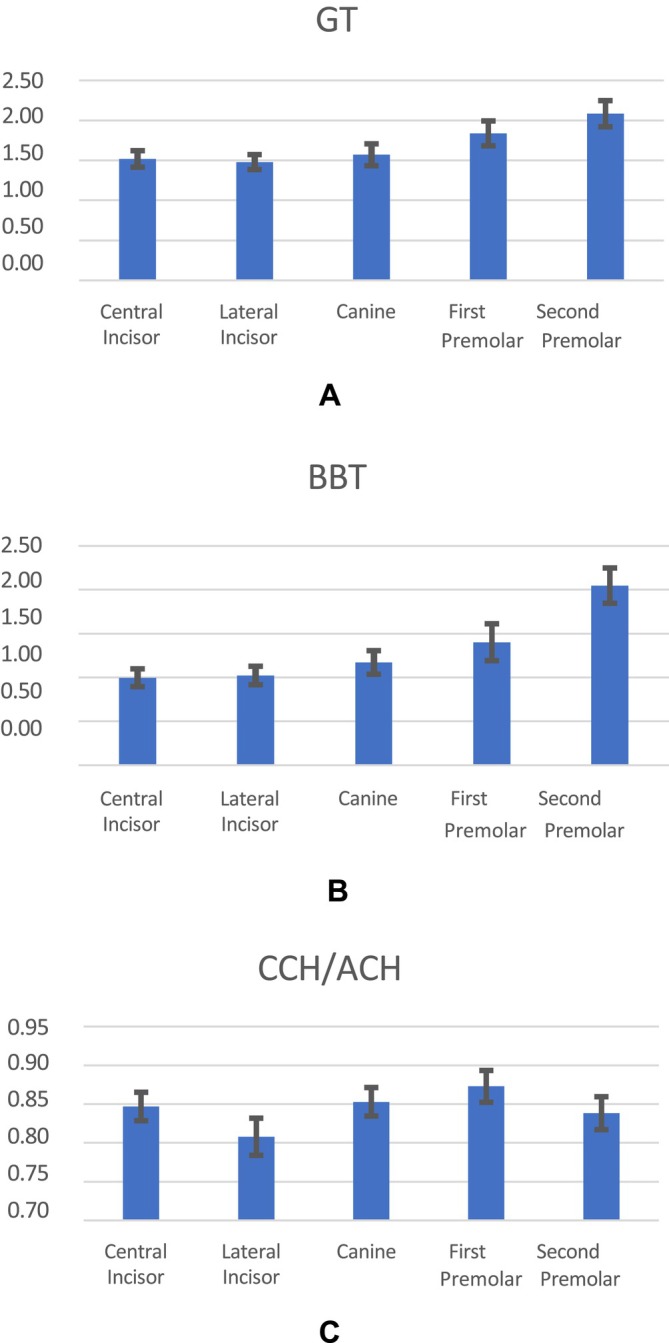
(A) Mean and SD of GT by tooth position. (B) Mean and SD of BBT by tooth position. (C) Mean and SD of CCH/ACH by tooth position. ACH: anatomical crown height. BBT: buccal bone thickness. CCH: clinical crown height. GT: gingival thickness.

The overall means, as well as the means for CI, LI, CA, P1, and P2 of GT, BBT, and CCH/ACH, are presented in (Figure 4 and Table 1). A statistically significant negative correlation of −0.29 (*p* < 0.001) and −0.17 (*p* = 0.01) between both GT and BBT, respectively, to age was found. No statistically significant difference was seen between males and females for GT or BBT. There was a statistically significant global difference between tooth positions for GT (*p* < 0.001), BBT (*p* < 0.001), and CCH/ACH (*p* = 0.001).

Both GT and BBT gradually increased from anterior to posterior direction. Specifically, for GT, there was a statistically significant pairwise difference between CI and P1 (*p* = 0.02), between CI and P2 (*p* < 0.001), between LI and P1 (*p* = 0.01) and P2 (*p* < 0.001) and between CA and P2 (*p* < 0.001). For BBT, there was a statistically significant pairwise difference between CI and P1 (0.004), between CI and P2 (*p* < 0.001), between LI and P1 (*p* = 0.01), between LI and P2 (*p* < 0.001), between CA and P2 (*p* < 0.001) and between P1 and P2 (*p* < 0.001). For CCH/ACH, there was a statistically significant pairwise difference between LI and CA (*p* = 0.02) and between LI and P1 (*p* = 0.001). The CCH/ACH ratio at LI was lower than at CA and P1.

**FIGURE 5:**
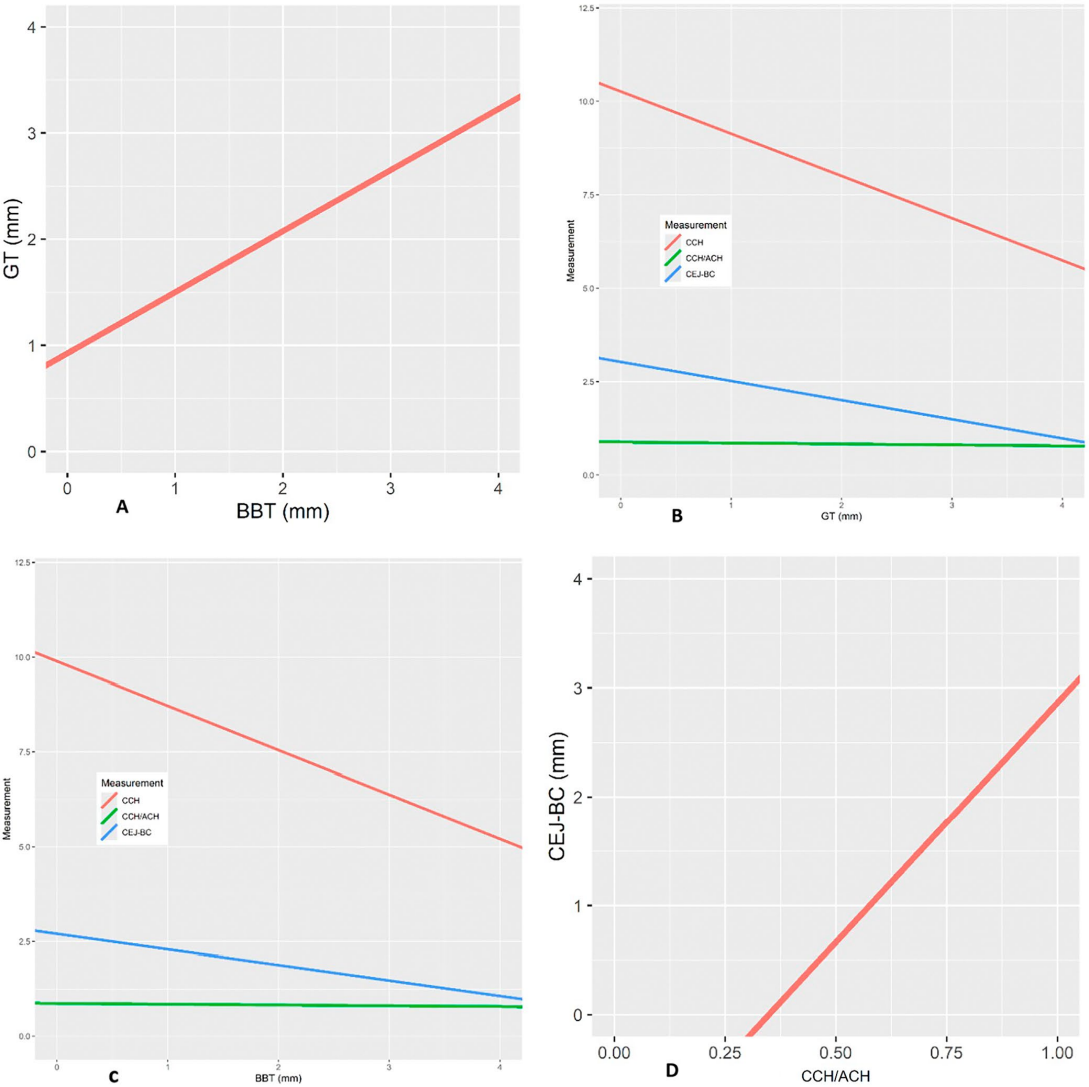
(A) Positive correlation between GT and BBT (0.72, *p* < 0.001). (B) Negative correlation between GT and CCH (−0.39, *p* < 0.001), CCH/ACH (− 0.17, *p* = 0.006), and CEJ‐BC (−0.32, *p* < 0.001). (C) Negative correlation between BBT and CCH (−0.50, *p* < 0.001), CCH/ACH (−0.18, *p* = 0.004) and CEJ‐BC (−0.32, *p* < 0.001). (D) Positive correlation between CEJ‐BC and CCH/ACH (0.41, *p* < 0.001). ACH: anatomical crown height. BBT: buccal bone thickness. BC: bone crest. CCH: clinical crown height. CEJ: cementoenamel junction. GT: gingival thickness.

A statistically significant positive correlation of 0.72 (*p* < 0.001) was found between GT and BBT (Figure 5A). There was a statistically significant negative correlation between GT and CCH (−0.39, *p* < 0.001), CCH/ACH (−0.17, *p* = 0.006) and CEJ‐BC (−0.32, *p* < 0.001) (Figure 5B).

There was a statistically significant negative correlation between BBT and CCH (−0.50, *p* < 0.001), CCH/ACH (−0.18, *p* = 0.004), and CEJ‐BC (−0.32, *p* < 0.001) (Figure 5C). A statistically significant positive correlation of 0.41 (*p* < 0.001) between CCH/ACH and the CEJ‐BC distance was found (Figure 5D).

## 4 Discussion

Although CBCT alone has commonly been used to assess gingival phenotype [14, 15, 16, 18, 19, 20, 21], studies have shown combining IOS and CBCT through the superimposition of STL and DICOM files is as reliable as the direct clinical approach using endodontic spreaders for measuring gingival thickness [24]. The accuracy and reliability of gingival thickness measurements improve when DICOM and STL files are superimposed, compared to using DICOM files alone, likely because STL files provide clearer reference outlines, enhancing the assessment of gingival tissues [25]. Superimposing multiple STL files onto DICOM files allows for the evaluation of the stability of gingival soft tissues over time and monitoring outcomes of soft tissue procedures [22].

According to the results of this investigation, gingival thickness and BBT were found to have a positive correlation, which is in agreement with the previous investigations [17, 18, 19, 20], which found a range of positive correlations between GT and BBT. Our value, however, is greater than the moderate correlations seen by Fu et al. and Younes et al. [18, 21] Differences in values may be due to both sample size and the differences in apico‐coronal references for GT and BBT.

Of note, is the significant negative correlation between both BBT and GT with age. Our findings align with other studies that have investigated BBT along the labial/buccal surface [26, 27, 28, 29], which can be attributed to age‐related alterations in bone metabolism, potentially leading to decreased bone mass and thickness in the buccal bone wall of anterior maxillary teeth [30]. Similarly, our findings agree with studies showing that gingival thickness tends to decrease with age. Younger individuals exhibit significantly thicker gingiva compared to older individuals, likely due to the densification of connective tissue and a reduction in cell count, resulting in thinner epithelium and diminished keratinization [31, 32].

By understanding the potential pre‐operative reasons for a more coronal position of the gingival margin, periodontists can improve their treatment outcomes. Coslet's and Ragghianti Zangrando's APE classifications describe both the classification of APE and approaches to treatment but fall short of addressing why this discrepancy in gingival margin position occurs [8, 9]. In cases diagnosed with APE subgroup B, the corono‐apical dimension of the gingiva is preserved despite a lack of cementum for connective tissue attachment; the gingiva is minimally attached to the cementum while junctional epithelium and sulcular epithelium are at the level of the enamel. This coronal position of the gingival margin is likely a result of the soft tissue preserving the predetermined soft tissue dimension, which is consistent with previous studies investigating supracrestal tissue attachment dimensions [17, 33]. The positive correlation found in our investigation between CCH/ACH and CEJ‐BC distance, along with the negative correlation between BBT and CEJ‐BEC, suggests that a thicker bone may prevent the crestal buccal bone from undergoing significant apical remodeling. This would position the osseous crest closer to the CEJ, leading to a more coronal gingival margin and shorter clinical crowns; this may explain the mechanism behind APE subgroup B.

On the other hand, the significant negative correlation for both GT and BBT to CCH/ACH indicates thicker buccal gingiva and bone result in a shorter clinical crown. This correlation may partially explain the coronal position of the buccal gingival margin in Coslet APE subgroup A despite the presence of a normal CEJ‐BC distance of 1.5–2 mm. This also implies that adjustment of bone and soft tissue thickness buccolingually may be beneficial when performing esthetic or restorative crown lengthening in lieu of a purely corono‐apical adjustment. The question remains, however, whether adjusting BBT alone would result in remodeling of soft tissue and a decrease in GT. Based on this finding, addressing only the vertical space between the CEJ and BC, without adjusting BBT, may hinder the desired apical displacement of the gingival margin in both esthetic and functional crown lengthening procedures.

Our results show a trend for increase in GT and BBT from anterior to posterior. The mean increase in BBT from maxillary CI to P2 was 1.05 mm. This agrees with Katranji et al. who found an increase in BBT from anterior to molar region in both the maxilla and mandible [34]. Katranji et al. found a mean average increase of 1.3 mm from anterior to posterior for maxilla and mandible; this however includes the molar areas. Tsigarida et al. similarly concluded in their systematic review that BBT tends to get thicker from anterior to posterior positions in the arch [35].

The use of digital technology, as demonstrated in our investigation and previous studies, may serve as a non‐invasive, reliable diagnostic tool for the preoperative assessment of gingival phenotype and bone thickness, as well as the diagnosis and treatment planning of esthetic crown lengthening procedures. It allows for precise preoperative evaluation of often overlooked factors, such as labial gingival and alveolar bone thicknesses and their relationship to the CEJ, which cannot be accurately captured using traditional techniques. This is especially valuable in cases with APE Subgroup A, where thick labial bone is often an intraoperative finding rather than a preoperative assessment with a predetermined treatment plan [22].

The limitations of this study include our relatively small cohort size due to the elimination of many screened subjects which did not meet the criteria for clear CBCT images and superimposition. Measurements were limited to the mid‐facial of each tooth, restricting applicability to interproximal sites. Unlike previous studies that measured labial bone thickness (BBT) at multiple fixed points [19, 20, 21, 26, 27, 28], we utilized the thickest portion of the buccal bone on the coronal half of the root to account for the varying configuration of the marginal bone in our subject pool. While this approach aimed to facilitate accurate representation of this parameter, it may have introduced variability in measurement location, thus affecting reproducibility compared to other studies. Additionally, conclusions regarding sex and race could not be drawn due to an imbalance in participant distribution across gender and demographic groups, as well as the small cohort size. Finally, age‐related findings should be interpreted with caution due to the unequal representation of age groups, highlighting the need for further research with larger, more diverse samples to validate these findings.

Despite the limitations, we believe the findings of our study might serve as a basis for future prospective studies with progressive enhancements in CBCT resolution and a larger sample size, ensuring equal representation of each individual tooth position. Integrating DICOM and STL into our protocol would contribute to the expanding knowledge base of modern digital technology, confirming their use as valuable clinical and research tools for measuring tooth and dentogingival dimensions. This finding is reinforced by previous investigations, including our own [17], consistently demonstrating the accuracy and non‐invasive nature of these digital tools.

## 5 Conclusions

Within the limitations of our investigation, this study concluded that gingival thickness and BBT are positively correlated. The gingival margin position in the esthetic zone is influenced by buccal gingival and bone thickness, BC‐CEJ distance, and tooth position. Thicker gingiva and buccal bone, along with a higher coronal bone crest, are associated with a more coronal gingival margin. Practitioners should consider these factors when diagnosing, planning, and treating teeth in the esthetic zone.

## Author Contributions

Conceptualization, study design, and literature review: Hesham H. Abdulkarim, Bryan Habet, and Alexander G Ahmadi. Data collection: Hesham H. Abdulkarim and Bryan Habet. Data curation: Hesham H. Abdulkarim. Formal analysis: Mary Ying‐Fang Wang. Figures: Elio Reyes Rosales, Bryan Habet, and Hesham H. Abdulkarim. All authors contributed to the manuscript, final edits and approved the final version of the manuscript.

## Ethics Statement

Approved by A. T. Still University Institutional Review (IRB) in December 2021 (IRB NA20221103‐001).

## Consent

Informed consents were obtained from all participants.

## Conflicts of Interest

The authors declare no conflicts of interest.

## Data Availability

The data that support the findings of this study are available on request from the corresponding author. The data are not publicly available due to privacy or ethical restrictions.

## References

[1] A. H. Tjan , G. D. Miller , and J. G. The , “Some Esthetic Factors in a Smile,” Journal of Prosthetic Dentistry 51, no. 1 (1984): 24–28, 10.1016/s0022-3913(84)80097-9. 6583388

[2] A. Volchansky and P. Cleaton‐Jones , “Clinical Crown Height (Length)–a Review of Published Measurements,” Journal of Clinical Periodontology 28, no. 12 (2001): 1085–1090, 10.1034/j.1600-051x.2001.281201.x. 11737504

[3] J. D. Sterrett , T. Oliver , F. Robinson , W. Fortson , B. Knaak , and C. M. Russell , “Width/Length Ratios of Normal Clinical Crowns of the Maxillary Anterior Dentition in Man,” Journal of Clinical Periodontology 26 (1999): 153–157. 10100040 10.1034/j.1600-051x.1999.260304.x

[4] C. S. Handelman , A. P. Eltink , and E. BeGole , “Quantitative Measures of Gingival Recession and the Influence of Gender, Race, and Attrition,” Progress in Orthodontics 19, no. 1 (2018): 5, 10.1186/s40510-017-0199-4. 29376198 PMC5787531

[5] G. V. Black , Descriptive Anatomy of the Human Teeth (Pennsylvania: SS White Manufacturing Company, 1897).

[6] B. Gottlieb , “Active and Passive Eruption of Teeth,” Journal of Dental Research 214 (1933): 214.

[7] H. M. Goldman and D. W. Cohen , Periodontal Therapy (St. Louis (MO): CV Mosby, 1968).

[8] J. G. Coslet , R. Vanarsdall , and A. Weisgold , “Diagnosis and Classification of Delayed Passive Eruption of the Dentogingival Junction in the Adult,” Alpha Omegan 70 (1977): 24–28. 276255

[9] M. S. Ragghianti Zangrando , G. F. Veronesi , M. V. Cardoso , et al., “Altered Active and Passive Eruption: A Modified Classification,” Clinical Advances in Periodontics 7 (2017): 51–56.

[10] J. Ainamo and H. Loe , “Anatomical Characteristics of Gingiva. A Clinical and Microscopic Study of the Free and Attached Gingiva,” Journal of Periodontology 37 (1966): 5–13. 4955513 10.1902/jop.1966.37.1.5

[11] A. C. Carbone , J. C. Joly , J. Botelho , et al., “Long‐Term Stability of Gingival Margin and Periodontal Soft‐Tissue Phenotype Achieved After Mucogingival Therapy: A Systematic Review,” Journal of Clinical Periodontology 51, no. 2 (2024): 177–195, 10.1111/jcpe.13900. 37963451

[12] E. D'Silva , D. Fraser , B. Wang , A. B. Barmak , J. Caton , and A. Tsigarida , “The Association Between Gingival Recession and Buccal Bone at Maxillary Anterior Teeth,” Journal of Periodontology 91 (2020): 484–492. 31512742 10.1002/JPER.19-0375

[13] D. M. Kim , S. H. Bassir , and T. T. Nguyen , “Effect of Gingival Phenotype on the Maintenance of Periodontal Health: An American Academy of Periodontology Best Evidence Review,” Journal of Periodontology 91, no. 3 (2020): 311–338, 10.1002/JPER.19-0337. 31691970

[14] J. Wang , S. Cha , Q. Zhao , and D. Bai , “Methods to Assess Tooth Gingival Thickness and Diagnose Gingival Phenotypes: A Systematic Review,” Journal of Esthetic and Restorative Dentistry 34, no. 4 (2022): 620–632, 10.1111/jerd.12900. 35297167

[15] B. S. de Freitas Silva , J. K. Silva , L. R. Silva , et al., “Accuracy of Cone‐Beam Computed Tomography in Determining Gingival Thickness: A Systematic Review and Meta‐Analysis,” Clinical Oral Investigations 27, no. 5 (2023): 1801–1814, 10.1007/s00784-023-04905-7. 36757462

[16] H. Gluckman , C. C. Pontes , J. Du Toit , C. Coachman , and M. Salama , “Dimensions of the Dentogingival Tissue in the Anterior Maxilla. A CBCT Descriptive Cross‐Sectional Study. Int,” Journal of Esthetic Dentistry 16, no. 4 (2021): 580–592. 34694081

[17] H. H. Abdulkarim , N. M. Antoine , M. Y. Wang , E. R. Rosales , and D. D. Miley , “Digital Assessment of Supracrestal Tissue Attachment and Its Correlation With Dentogingival Components,” Clinical Advances in Periodontics (2024), 10.1002/cap.10280. PMC11718352 38348934

[18] J. H. Fu , C. Y. Yeh , H. L. Chan , N. Tatarakis , D. J. Leong , and H. L. Wang , “Tissue Biotype and Its Relation to the Underlying Bone Morphology,” Journal of Periodontology 81, no. 4 (2010): 569–574, 10.1902/jop.2009.090591. 20367099

[19] E. Couso‐Queiruga , E. P. Barboza , G. Avila‐Ortiz , O. Gonzalez‐Martin , L. Chambrone , and D. M. Rodrigues , “Relationship Between Supracrestal Soft Tissue Dimensions and Other Periodontal Phenotypic Features: A Cross‐Sectional Study,” Journal of Periodontology 94, no. 8 (2023): 944–955, 10.1002/JPER.22-0434. 36797817

[20] H. Zhao , L. Zhang , H. Li , A. Hieawy , Y. Shen , and H. Liu , “Gingival Phenotype Determination: Cutoff Values, Relationship Between Gingival and Alveolar Crest Bone Thickness at Different Landmarks,” Journal of Dental Sciences 18 (2023): 1544–1552. 37799899 10.1016/j.jds.2023.03.003 PMC10547992

[21] F. Younes , A. Eghbali , M. Raes , T. De Bruyckere , J. Cosyn , and H. De Bruyn , “Relationship Between Buccal Bone and Gingival Thickness Revisited Using Non‐Invasive Registration Methods,” Clinical Oral Implants Research 27 (2016): 523–528. 26010518 10.1111/clr.12618

[22] H. H. Abdulkarim , A. Vij , and D. E. McLeod , “Combination Scan Technique: An Innovative Approach to Diagnosing Altered Passive Eruption,” Journal of Cosmetic Dentistry 36, no. 3 (2020): 40.

[23] C. Mangano , F. Luongo , M. Migliario , C. Mortellaro , and F. G. Mangano , “Combining Intraoral Scans, Cone Beam Computed Tomography and Face Scans: The Virtual Patient,” Journal of Craniofacial Surgery 29 (2018): 2241–2246. 29698362 10.1097/SCS.0000000000004485

[24] E. Couso‐Queiruga , M. Tattan , U. Ahmad , C. Barwacz , O. Gonzalez‐Martin , and G. Avila‐Ortiz , “Assessment of Gingival Thickness Using Digital File Superimposition Versus Direct Clinical Measurements,” Clinical Oral Investigations 25, no. 4 (2021): 2353–2361, 10.1007/s00784-020-03558-0. 32865627

[25] E. Couso‐Queiruga , C. Raabe , U. C. Belser , et al., “Non‐Invasive Assessment of Peri‐Implant Mucosal Thickness: A Cross‐Sectional Study,” Journal of Periodontology 94, no. 11 (2023): 1315–1323, 10.1002/JPER.23-0102. 37332251

[26] J. Gakonyo , A. J. Mohamedali , and E. K. Mungure , “Cone Beam Computed Tomography Assessment of the Buccal Bone Thickness in Anterior Maxillary Teeth: Relevance to Immediate Implant Placement,” International Journal of Oral & Maxillofacial Implants 33 (2018): 880–887. 30025005 10.11607/jomi.6274

[27] V. Braut , M. M. Bornstein , U. Belser , and D. Buser , “Thickness of the Anterior Maxillary Facial Bone Wall—A Retrospective Radiographic Study Using Cone Beam Computed Tomography,” International Journal of Periodontics & Restorative Dentistry 31 (2011): 125–131. 21491011

[28] H. M. Wang , J. W. Shen , M. F. Yu , X. Y. Chen , Q. H. Jiang , and F. M. He , “Analysis of Facial Bone Wall Dimensions and Sagittal Root Position in the Maxillary Esthetic Zone: A Retrospective Study Using Cone Beam Computed Tomography,” International Journal of Oral & Maxillofacial Implants 29 (2014): 1123–1129. 25216138 10.11607/jomi.3348

[29] J. G. Dos Santos , A. P. Oliveira Reis Durao , A. C. de Campos Felino , C. Lobo , and R. M. de Faria de Almeida , “Analysis of the Buccal Bone Plate, Root Inclination and Alveolar Bone Dimensions in the Jawbone: A Descriptive Study Using Cone‐Beam Computed Tomography,” Journal of Oral & Maxillofacial Research 10 (2019): 10. 10.5037/jomr.2019.10204 PMC6683387 31404187

[30] X. Wang , X. Hu , H. Zhang , H. Zhang , and Z. Song , “Analysis of the Dimensions of Buccal and Palatal Bone Wall in the Maxillary Anterior Esthetic Zone: A Cone‐Beam Computed Tomography Study,” Research Square (2022), 10.21203/rs.3.rs-2230311/v1.

[31] K. L. Vandana and B. Savitha , “Thickness of Gingiva in Association With Age, Gender and Dental Arch Location,” Journal of Clinical Periodontology 32, no. 7 (2005): 828–830, 10.1111/j.1600-051X.2005.00757.x. 15966893

[32] R. Kolte , A. Kolte , and A. Mahajan , “Assessment of Gingival Thickness With Regards to Age, Gender and Arch Location,” Journal of Indian Society of Periodontology 18, no. 4 (2014): 478–481, 10.4103/0972-124X.138699. 25210263 PMC4158590

[33] A. W. Gargiulo , F. M. Wentz , and B. Orban , “Dimensions and Relations of the Dentogingival Junction in Humans,” Journal of Periodontology 32 (1961): 261–267.

[34] A. Katranji , K. Misch , and H. L. Wang , “Cortical Bone Thickness in Dentate and Edentulous Human Cadavers,” Journal of Periodontology 78 (2007): 874–878. 17470021 10.1902/jop.2007.060342

[35] A. Tsigarida , J. Toscano , B. B. De Brito , et al., “Buccal Bone Thickness of Maxillary Anterior Teeth: A Systematic Review and Meta‐Analysis,” Journal of Clinical Periodontology 47 (2020): 1326–1343. 32691437 10.1111/jcpe.13347

